# The bright side of supplier concentration: Investor attitudes towards the reopening policy in China

**DOI:** 10.1371/journal.pone.0313682

**Published:** 2024-11-18

**Authors:** Jie Su

**Affiliations:** School of Statistics and Management, Shanghai University of Finance and Economics, Shanghai, China; Ataturk University, TÜRKIYE

## Abstract

Supplier concentration (SUC) has disadvantage of vulnerability along with cost savings and efficiency. While current scholarship focus on the vulnerability of firms with centralized suppliers during the COVID-19 epidemic, there is no empirical study that explores the impact of post-disaster SUC on firm value as countries removing regional isolation policy. I focus on the impact of COVID-19 reopening policy on investor attitudes towards SUC after the resolution of a supply chain disruption crisis. I try to examine whether investors still perceive SUC as a risk signal or as a positive signal for rapid recovery. Using the event shock of China’s reopening announcement and data on A-share listed companies, I find that SUC has a positive impact on cumulative abnormal returns at reopening. I also find that positive effect of SUC is more prominent for firms that benefit from a larger reduction in transaction costs due to the reopening policy. I also analyze the moderating effect and find that information intermediaries such as analysts and media attention amplify the positive effects of SUC. My research provides new perspective on achieving post-disaster value enhancement through SUC.

## 1. Introduction

Supplier allocation—diversification or concentration—is a key consideration for corporate sustainability. Supplier concentration (SUC) refers to a company’s reliance on a small number of suppliers, which reduces transaction costs, improves coordination, and increases operational efficiency [1,2]. Companies that focus on SUC benefit from closer, more stable relationships with suppliers, which contribute to smoother production and optimized inventory management [3–5]. However, centralized suppliers also increase a firm’s vulnerability to disruptions, as problems with one supplier can severely affect the production operations of the focal firm [6]. Reducing concentration in favor of cooperation with multiple decentralized suppliers is called supplier diversification (SUD). SUD is more resilient to external shocks such as natural disasters or market disruptions, but is usually accompanied by higher transaction costs and operational complexity [7–9]. Balancing these opposing strategies is a long-term challenge in supply chain management since SUC not only affects a firm’s capital structure, innovation capability, production costs, and operating performance at the operational level, but also triggers volatility in stock and bond prices [2,6,10–17].

COVID-19 pandemic has brought the risks and benefits of supplier-customer relationships to the forefront of attention [8,10,18,19]. Studies such as Lin et al. (2020) have shown that SUD stabilizes the supply chain and improves profitability during pandemic disruptions [8]. Meanwhile, Cheng et al. (2022), using the Wuhan lockdown as a case study, found that the stock prices of companies with concentrated suppliers dropped sharply due to investor concerns about supply chain risks [10]. These findings suggest that SUC can amplify the risk of disruption to firms during a crisis, especially when faced with a lockdown measure and isolation policy. However, as the epidemic subsided and countries gradually lifted strict isolation policies, the risk of supply chain disruptions gradually declined. The question that arises is whether investors will still see SUC as a negative signal after the disaster is over, as they did at the beginning of the epidemic [10]? Or will investors recognize its potential benefits during the recovery?

As a result of recurring outbreaks, some countries have relaxed parts of their lockdown restrictions during periods of a lower disease count, but have not announced a complete end to controls [20–22]. Supply chain risks associated with SUC were reduced to moderate degrees during this period. The literature has partly considered both the disruption period and the temporary recovery period to analyze the dynamics of SUC affecting the production status of firms. Scholars such as Jiang et al. (2023) and Polyviou et al. (2023) investigate how supplier concentration affects firms’ resilience and productivity during a short recovery period [20,21]. Their findings highlight a subtle point: SUC may help firms survive crises by increasing resilience and mitigating the damage of supply disruptions during disruptions, however, it may lead to the opposite result during partial recoveries. These studies suggest that SUC is not a universally beneficial strategy, especially as markets transition from crisis to recovery.

The end of the epidemic has changed the supply chain in at least two ways. First, unlike the epidemic period, the lockdown and isolation policies that posed the greatest threat to SUC have completely disappeared since the reopening was announced. This period is a very different environment from the temporary recovery previously studied, as companies are now able to operate without fear of a new round of restrictions or supply disruptions. Second, the post-disaster rebound in the consumer market and the lack of supply inventories created a large supply-demand gap that required firms to respond in a timely manner [23]. By spending a lot of time and money on transactions, SUC makes it easier for firms to form closely coordinated relationships with key suppliers, which improves the efficiency of their response to the market [24]. Therefore investors’ perception of SUC may also change. However, existing research is inconclusive as to whether SUC affects the value of firms after a disaster, which is an open question. Unexpected disasters similar to COVID-19 can still occur at any time in the future, and how to prevent disasters before they occur is equally important as how to recover from them [25]. Therefore, it is necessary to analyze the impact of post-disaster SUC on business value.

The announcement of China’s reopening provides the appropriate conditions for examining this issue. on December 27, 2022, China announced the reclassification of COVID-19 from Category A disease to Category B disease, which completely declared the end of the era of lockdown and isolation. To deepen our understanding of the supply chain relationship, I analyze the impact of post-disaster SUC on stock values using China’s reopening announcement as an event shock. I find that SUC is positively related to the cumulative abnormal returns of stocks, validating the confidence of stock investors in firms with high SUC after the disaster.

This study contributes to the literature in two key areas. First, it adds to the understanding of post-crisis impacts. Existing studies have investigated market reactions to supply chain disruptions during crises, yet investor confidence in post-disaster firm reconstruction is important and has lacked attention. This study is one of the first papers to explore the impact of reopening policy shocks. Second, it contributes to the understanding of the impact of supplier relationships, a topic that is continuously debated in the economic literature. My empirical results show the bright side of SUC and validate investor optimism during reopening periods. This provides new theoretical insights into how high SUC firms can achieve sustainable growth and competitive advantage in a fully reopened economy.

## 2. Literature review and hypothesis

### 2.1. Literature review

Suppliers and customers form close relationships through purchases and sales, and when one side of the supply chain is shocked the other side also triggers stock price volatility. Research has shown that investors view large suppliers and customers during the contract period as communities of interest, and quarterly surplus announcements from customers can trigger changes in suppliers’ stock prices through information spillovers [26]. When one firm’s financial performance is poor, the other firm’s stock price can also experience negative volatility [27,28]. However, there are still fewer analyses on the impact of SUC on stock prices.

There are two typical views on the impact of SUC on enterprises: value creation and value plunder. The cooperation view holds that suppliers have a significant position as the upstream of the industrial chain, and centralized procurement is conducive to the establishment of strategic cooperative relationships, thereby increasing enterprise value. Organizational complexity is an important source of inefficiency. Whether the complexity comes from upstream suppliers, downstream customers, or the firm itself, it can be harmful to performance [29]. With the specialization of products, firms tend to specialize in one production chain and achieve economies of scale by centralizing the supply chain, thereby increasing production efficiency, improving financial performance and enhancing risk resistance [3,4,11,30]. Li et al. found that manufacturers acquire hard-to-imitate tacit knowledge and high levels of explicit knowledge through interactions with key suppliers, which help firms gain an advantage in product competition [15]. In terms of financing constraints, supplier relationships facilitate firms’ access to bank loans and commercial credit financing, saving them from performance decline during economic downturns [31,32]. Cen et al. found that relying on large suppliers can also help firms achieve tax avoidance through, for example, tax burden shifting [33]. Due to the positive effect of centralized supply chain relationships on operational efficiency, Patatoukas finds that holding stocks with high customer concentration earns additional returns [34].

The value-grabbing perspective argues that centralized purchasing may lead to an inelastic supply chain that is prone to opportunism and supply chain risk [16,35]. High SUC can expose a firm to operational vulnerabilities, especially during external shocks. A limited number of suppliers can lead to an inflexible supply chain, making firms more vulnerable to supply chain disruptions caused by events such as geopolitical instability or economic downturns [8,20,36]. For example, during the COVID-19 pandemic, firms with high SUCs were at significant risk of supply disruptions, which negatively impacted their stock performance [10]. Dependence on a small number of suppliers makes firms vulnerable to bargaining power imbalances, and suppliers may demand more favorable terms, thereby reducing the firm’s financial flexibility [16]. In addition, companies typically have to hold large precautionary cash reserves to offset the risk of supply chain disruptions, thereby limiting the capital available for other growth opportunities [12,37]. External shocks, such as natural disasters or pandemics, can amplify these risks and lead to negative investor reactions, especially during periods of high uncertainty [10].

In summary, the literature provides arguments for the benefits and risks of SUC on firms’ financial performance, but gaps in insights for the stock market remain. The existing literature on SUC focuses on the dual nature of SUC, exploring its benefits in terms of fostering strategic relationships and economies of scale, as well as its risks in terms of supply chain fragility and reduced flexibility during crises. However, while most studies have emphasized these dynamics in times of economic instability or crisis, with particular emphasis on the negative impact of SUC during the COVID-19 pandemic and other external shocks, they have not fully explored the role of SUC in the post-crisis recovery phase [8,10,20–22]. By shifting the focus to the post-disaster context, this paper examines, in particular, how investor perceptions of SUC evolve as external risks recede.

### 2.2. Hypothesis development

COVID-19 exploded in Wuhan, China, in 2019, and with it came home office and logistical disruptions due to strict regional mobility restrictions. COVID-19 had a dramatic impact on supply chains, and much of the literature discusses the risks of centralized supplier dependence. The cessation of supply from an upstream supplier directly leads to leads to a downstream customer’s production decline or even shutdown [38]. Centralized procurement of goods or services led to firms suffering more stock price declines during the epidemic, especially for those located in the hardest-hit areas of the epidemic [10,39]. In contrast, firms that maintained lower SUC before the epidemic or developed local suppliers with lower costs after the outbreak achieved better financial performance [40]. Affected by the disaster, investors continue to be strongly risk averse for an extended period of time, and it may take a long time for this risk aversion to return to pre-disaster levels [41–43]. During the three-year-long epidemic, the unpredictable recurrence of the epidemic created uncertainty about economic activity, and this uncertainty amplified investors’ risk aversion [44]. As a result, after the reopening investors may still insist on perceiving SUC as a risk signal, thus implementing this perception into stock trading and ultimately leading to a decline in stock prices.

However, SUC is also likely to have value-added effects on firms under the shock of reopening. First, with the announcement of the reopening policy, the risk of supply chain disruptions, which investors were concerned about, was substantially reduced. One of the most important triggers of supply chain risk is uncertainty in the external environment [45]. Market demand, transportation speed, and human capital during an epidemic are highly unpredictable as they are affected by epidemic prevention and control policies. The risk of supply chain disruptions due to environmental uncertainty is higher during epidemics, thus negatively affecting firms with high SUC [10]. However, with the reopening policy announcing the removal of strict regional mobility restrictions, the uncertainty of the external environment is subsequently reduced, and firm-to-firm exchanges, production, and transportation links are no longer subject to mandatory restrictions.

Second, during the economic recovery phase, a concentration of suppliers may help firms to realize economies of scale by lowering transaction costs and improving the efficiency of cooperation. Transaction cost theory suggests that a small and concentrated number of suppliers has advantages in reducing transaction costs, such as reducing the cost of adjusting inventory and communication [3–5]. Through high-intensity communication and cooperation, firms and suppliers may form closer economic relationships. When firms anticipate a higher probability of recovery in future economic conditions, then the expected high returns from cooperation will prevent suppliers and customers from engaging in opportunistic behavior [46,47]. After the end of the epidemic, market consumption may surge in a short period of time, and companies need to respond quickly to market changes to fulfill demand [23]. Collaboration with suppliers at this time facilitates the adjustment of production structure, which leads to expansion of production scale and rapid capture of market share [48]. Thus, SUC may have value-added effects on firms after reopening.

Third, based on the efficient market hypothesis, stock markets are able to respond in a timely manner to information that affects firm value. Supply chain information is frequently used in stock price valuation, especially after the global supply chain disruptions caused by the epidemic, when investor attention to the resilience of upstream and downstream supply chains reached new heights [10,38,49,50]. For example, those firms with a high concentration of suppliers at the time of the announcement of the lockdown experienced a sharper downward movement in their stock prices due to concerns about future uncertainty [10]. Similarly, as the reopening policy releases clear signals of an end to the lockdown and isolation, investors are likely to realize the value of SUC in the light of shifting economic conditions and practice this perception in stock prices. As a result, reopening shocks may lead to upward movement in the stock prices of firms with high SUC.

Based on the above analysis, I argue that stock investors in reopening shocks may view SUC as a signal of risk or as a signal of value. On the one hand, after a major supply chain disruption risk, investors may still be suffering from the trauma of supply chain risk and thus sell stocks with high SUC. On the other hand, working with centralized suppliers may benefit firms in gaining rapid growth after the outbreak, thus raising stock prices. Therefore, I propose the following competing hypothesis:

Hypothesis H1a: SUC has a negative effect on cumulative abnormal returns under reopening shocks.
Hypothesis H1b: SUC has a positive effect on cumulative abnormal returns under reopening shocks.

## 3. Data and methodology

### 3.1. Data sources

This study retrieve financial and stock return data from the China Stock Market & Accounting Research (CSMAR) database. The sample selection process is as follows: 1) The initial sample contains all A-share companies listed on the Shanghai and Shenzhen stock exchanges. 2) Financial sector companies were excluded due to their distinct accounting standards and regulatory environment, which differ significantly from non-financial firms. Financial firms accumulate value based on interest rates and regulatory factors rather than operational factors like supply chain management, which is the focus of this study. 3) Companies with stock short name labeled ST or *ST were excluded because they have financial problems. 4) Excludes companies that did not disclose the proportion of supplier purchases in their 2021 annual reports. Since the event date was December 27, 2022, and the annual reports for the year 2022 had not been published at that time, I chose financial data from 2021. 5) Stocks that are suspended during the event window were excluded. 6) Data with missing control variables were excluded. The final dataset contains 4043 samples.

### 3.2. Measurement of cumulative abnormal returns

Cumulative abnormal returns (CAR) is the dependent variable. Following Cheng et al (2022), I estimate CAR1 using a market model as follows [10]:

$$R_{i,t}=\alpha_{i}+\beta_{i}R_{m,t}+\varepsilon ,t\in (-210,-10),$$ (1)

where R_i,t_ denotes the return of stock i at date t. R_m,t_ denotes the market capitalization-weighted average return of all stocks in the entire Shanghai and Shenzhen A-share markets at date t, taking into account the reinvestment of cash dividends. t = 0 is the event day, which set as December 27, 2022, the first trading day after China announces the reopening measures. Following existing research, I choose the estimation window from 210 to 10 trading days prior to the event date. CAR1 is estimated as follows:

$$CAR1_{i}[t_{1},t_{2}]=\sum_{t_{1}}^{t_{2}}AR_{i,t}=\sum_{t_{1}}^{t_{2}}\left(R_{i,t}-{\hat{\alpha }}_{i}-{\hat{\beta }}_{i}R_{m,t}\right),$$ (2)

where ${\hat{\alpha }}_{i}$ and ${\hat{\beta }}_{i}$ are estimated using Eq (1), CAR1[–1,1] and CAR1[–1,7] are the focus of this study. Higher CAR1 indicates more positive investor attitudes towards the stock, whereas lower CAR1 indicates more negative attitudes.

To ensure the robustness of the results, referring to the existing literature I also calculated CAR2 using the FAMA three-factor model by Eqs (3) and (4) [51–53].

$$R_{i,t}-R_{f,t}=\alpha_{i}+\beta_{1}(R_{m,t}-R_{f,t})+\beta_{2}SMB_{t}+\beta_{3}HML_{t}+\varepsilon ,t\in (-210,-10),$$ (3)

$$CAR2_{i}[t_{1},t_{2}]=\sum_{t_{1}}^{t_{2}}AR_{i,t}=\sum_{t_{1}}^{t_{2}}\left(R_{i,t}+R_{f,t}-{\hat{\alpha }}_{i}-{\hat{\beta }}_{1}(R_{m,t}-R_{f,t})-{\hat{\beta }}_{2}SMB_{t}-{\hat{\beta }}_{3}HML_{t}\right),$$ (4)

where *R*_f,*t*_ denotes the risk-free rate of return on day t, *SMB*_*t*_ is the market capitalization factor and *HML*_*t*_ is the book-to-market ratio factor. The factors *R*_f,*t*_, *SMB*_*t*_ and *HML*_*t*_ were obtained from the CSMAR database. ${\hat{\alpha }}_{i}$, ${\hat{\beta }}_{1}$, ${\hat{\beta }}_{2}$ and ${\hat{\beta }}_{3}$ are estimated using Eq (3), CAR2[–1,1] and CAR2[–1,7] are the focus of this study.

### 3.3. Measurement of supplier concentration

The independent variable, Supplier Concentration (SUC), is measured following Cheng et al (2022) and Zhang et al (2020) [10,12], defined as the summation of transaction proportions among the top five suppliers:

$$SUC_{i}=\sum_{1}^{5}\omega_{i,j},$$ (5)

where *ω*_*i*,*j*_ represents the proportion of the total annual procurement amount from supplier j.

### 3.4. Empirical model

I use event study methodology to analyze the impact of SUC on market reactions. The empirical model is constructed as follows:

$$CAR_{i}[t_{1},t_{2}]=\alpha +\beta SUC_{i}+\sum \lambda_{j}Control_{i,j}+\sum \gamma_{k}Location_{k}+\sum \eta_{l}Industry_{l}+\mu ,$$ (6)

where *CAR*_*i*_[*t*_1_, *t*_2_] is cumulative abnormal returns of firm *i* in the event window [t_1_,t_2_]. I use *CAR*1_*i*_[*t*_1_, *t*_2_] and *CAR*2_*i*_[*t*_1_, *t*_2_] to denote CAR respectively. *SUC*_*i*_ denotes the supplier concentration of firm i in 2021. *Control*_*i*,*j*_ denotes a set of control variables for firm *i* to account for potential influences on stock performance. These control variables include asset size (SIZE), leverage ratio (LEV), book-to-market ratio (BM), Tobin’s Q value (TBQ), proportion of shares held by the top ten largest shareholders (TOP10), stock volatility (VOL), institutional investor shareholding (Inhold), and disclosure of the specific name of top five suppliers (DISC). The financial variables in the control variables, except for VOL and DISC, are values for the third quarter of 2022, due to the fact that the annual report for 2022 had not yet been released at the event date. While SUC and DISC are values from the 2021 annual report because information on vendor transactions is only disclosed in the annual report. Additionally, I incorporate industry and location fixed effects to control for unobservable industry-specific and region-specific factors. The industry classification uses the latest 2012 Securities and Exchange Commission standards, with manufacturing industries subdivided into two-digit industries and the remaining industries subdivided into broad categories. Location classification uses two-digit administrative codes, i.e., provinces. To mitigate the potential influence of outliers, all continuous variables are winsorized at the 1st and 99th percentiles. Table 1 provides specific definitions for all variables.

**Table 1:** Variables definitions.

Variable	Measurement
CAR1	Cumulative abnormal returns, calculated using the market model. See Eqs (1) and (2) in the main text for specific calculations. The main event windows of focus are CAR1[–1,1] and CAR1[–1,7], with CAR1[0,1] and CAR1[–1,10] of focus in the robustness tests.
CAR2	Cumulative abnormal return, calculated using the FAMA three-factor model. The main event windows of focus are CAR2 [–1,1] and CAR2 [–1,7]. See Eqs (3) and (4) in the main text for specific calculations, with CAR2[0,1] and CAR2[–1,10] of focus in the robustness tests.
SUC	Supplier concentration, the proportion of purchases from the top five suppliers, with the specific calculation formula given in Eq (5) in the main text.
SIZE	Asset size, calculated as the logarithm of the firm’s total assets.
LEV	Leverage ratio, the ratio of total liability to total assets.
BM	Book-to-market value, the ratio of book value per share to the stock price per share.
TBQ	Torbin’s Q value, the ratio of market value to total assets.
TOP10	Percentage of shares held by the top ten largest shareholders.
VOL	Stock volatility, equal to the standard deviation of stock prices from 40 days prior to the event date to the previous ten trading days.
Inhold	Institutional investor shareholding.
DISC	Disclosure of the specific name of top five suppliers, equal to 1 if the specific name of at least one of the top five suppliers is published in the annual report, otherwise 0.
Season1_FAI	Fixed asset investment in the first quarter after reopening, equals the difference between cash paid to construct fixed assets and cash received from disposal of fixed assets divided by total assets.
Semi_FAI	Fixed asset investment in the second quarter after reopening, equals the difference between cash paid to construct fixed assets and cash received from disposal of fixed assets divided by total assets.
CAR3	Cumulative abnormal returns, calculated using the FAMA five-factor model. The main focus is on event windows of [–1,1] and [–1,7].
CAR1_IMP	Cumulative abnormal returns calculated using the market model with the policy implementation date as the event date. See Eqs (1) and (2) in the main text for specific calculations. The main event windows of interest are CAR1_IMP [–1,1] and CAR1_IMP [–1,7].
CAR2_IMP	Cumulative abnormal returns calculated using the FAMA three-factor model with the policy implementation date as the event date. See Eqs (1) and (2) in the main text for specific calculations. The main event windows of interest are CAR2_IMP [–1,1] and CAR2_IMP [–1,7].
AP	Accounts payable, equal to accounts payable divided by total assets.
FAR	Fixed asset ratio, equal to fixed asset divided by total assets.
CUC	Customer concentration, the proportion of sales to the top five customers.
Analyst	Analyst attention, equal to the natural logarithm of the number of analysts tracked plus one.
Media	Media attention, equal to the natural logarithm of the number of financial media reports appearing plus one.

## 4. Empirical results

### 4.1. Descriptive statistics

Table 2 provides descriptive statistics of the key variables. As shown in Table 2, when the reopening policy is announced, the minimum and maximum values of 3-day cumulative abnormal returns CAR1[–1,1] (CAR2[–1,1]) are -0.112 (-0.105) and 0.150 (0.159), respectively, and the minimum and maximum values of 9-day cumulative abnormal returns CAR1[–1,7] (CAR2[–1,7]) are -0.171 (-0.167) and 0.206 (0.208), implying that there is a large variation in the market response to reopening across stocks. The mean values of SUC is 0.351, implying that on average, listed companies get 35.1% of their production raw materials from the important five suppliers. Among the control variables, the mean value of DISC is 0.13, which indicates that only 13.2% of the companies publish the specific names of top five suppliers instead of using vague substitutes such as "Supplier 1". Other variables are within reasonable limits.

**Table 2:** Summary statistics.

	(1)	(2)	(3)	(4)	(5)	(6)
VarName	N	SD	Mean	Min	Median	Max
CAR1[–1,1]	4043	0.041	-0.005	-0.112	-0.009	0.150
CAR2[–1,1]	4043	0.041	0.001	-0.105	-0.003	0.159
CAR1[–1,7]	4043	0.062	-0.002	-0.171	-0.007	0.206
CAR2 [–1,7]	4043	0.061	-0.001	-0.167	-0.005	0.208
SUC	4043	0.196	0.351	0.057	0.309	0.917
SIZE	4043	1.295	22.392	20.151	22.170	26.425
LEV	4043	0.203	0.414	0.058	0.404	0.935
TOP10	4043	0.156	0.571	0.226	0.574	0.905
BM	4043	0.252	0.657	0.141	0.657	1.246
TBQ	4043	1.097	1.871	0.803	1.522	7.097
VOL	4043	0.010	0.024	0.009	0.022	0.058
Inhold	4043	0.244	0.395	0.006	0.393	0.909
DISC	4043	0.337	0.130	0.000	0.000	1.000

### 4.2. Baseline regression

Table 3 reports the effect of SUC on cumulative abnormal returns under reopening shocks. In columns (1) and (2), cumulative abnormal returns (CAR1) computed using the market model are the explanatory variables, and in columns (3) and (4), cumulative abnormal returns (CAR2) computed using the FAMA three-factor model are the explanatory variables. The event window for columns (1) and (3) is [–1,1], while columns (2) and (4) are expanded to [–1,7]. The results show that the coefficients of SUC are all significantly positive at least at the 5% level, indicating that the higher the SUC of the firm, the more positive the investor response to reopening shock. In terms of economic significance, for example, a one standard deviation increase in SUC in column (1) increases CAR by about 4.3% standard deviations (0.009 * 0.196 / 0.041 = 0.043). Overall, Table 3 validates Hypothesis H1b. While Cheng et al. suggests that investors may have reacted negatively to SUC at the beginning of the epidemic due to the potential risk of supply chain disruptions[10], the results in Table 3 indicate that investors had exact opposite reaction to SUC when the reopening was announced. Table 3 suggests that, after resolving the supply chain disruption crisis caused by COVID-19, investors had more confidence in companies with high SUC. Consequently, SUC is considered as positive signal.

**Table 3:** Baseline regression.

	(1)	(2)	(3)	(4)
	CAR1[–1,1]	CAR1[–1,7]	CAR2[–1,1]	CAR2[–1,7]
SUC	0.009***	0.009**	0.009***	0.009**
	(3.04)	(2.58)	(2.94)	(2.40)
SIZE	0.006***	0.004***	0.003***	0.003***
	(6.81)	(5.97)	(3.96)	(4.25)
LEV	0.006***	0.003	0.009***	0.006
	(3.31)	(0.60)	(4.84)	(1.35)
TOP10	0.023***	0.045***	0.021***	0.042***
	(3.86)	(4.17)	(3.46)	(4.02)
BM	-0.028***	-0.047***	-0.027***	-0.037***
	(-7.04)	(-6.18)	(-6.64)	(-5.02)
TBQ	0.003**	-0.002	0.002*	-0.002
	(2.52)	(-1.43)	(1.70)	(-1.31)
VOL	-0.250**	-0.406***	-0.215*	-0.431***
	(-2.37)	(-3.53)	(-1.97)	(-3.82)
Inhold	-0.008*	-0.021***	-0.008*	-0.020***
	(-1.79)	(-2.91)	(-1.76)	(-2.79)
DISC	0.002	-0.004	0.002	-0.004
	(1.13)	(-1.63)	(1.06)	(-1.52)
Constant	-0.129***	-0.080***	-0.072***	-0.054***
	(-6.39)	(-4.40)	(-3.43)	(-3.10)
Location FE	YES	YES	YES	YES
Industry FE	YES	YES	YES	YES
N	4043	4043	4043	4043
R2	0.130	0.133	0.119	0.118

Note:

*, **, *** denote significance at the 10%, 5%, and 1% levels, respectively; The robust standard errors shown in parentheses are clustered at the location level.

### 4.3. Robustness tests

#### 4.3.1 Supplier concentration and post-disaster recovery

Baseline regression examine the effect of SUC on firm value under reopening shocks using CAR. Moreover, if post-disaster SUC enhances firm value as investors expect, then I expect SUC to accelerate firms’ post-disaster recovery as well. Accelerator theory suggests that when firms expect an increase in market demand, they will invest more in fixed assets in order to expand production capacity and productivity [54]. Therefore, I expect SUC to boost firms’ fixed asset investment (FAI) in 2023.

To verify this expectation, I measure FAI using net cash invested in fixed assets divided by total assets and examine the impact of SUC on FAI. Specifically, I use the financial data of the first quarter and semiannual report of 2023 to calculate Season1_FAI and Semi_FAI, respectively, and use Season1_FAI and Semi_FAI to replace the explanatory variables in Model (6) for re-estimation, respectively. The other variables are consistent with model (6).

Table 4 shows that the coefficients of SUC in columns (1) and (2) are 0.004 and 0.01, respectively, and both are significant at the 1% level. Table 4 implies that the Season1_FAI and Semi_FAI increase by 7.1% and 8.9% standard deviations respectively as the SUC increases by one standard deviation. This suggests that SUC is significantly positively correlated with FAI in both first quarterly and semiannual reports after the reopening, which is consistent with the previous expectation.

**Table 4:** Impact of SUC on fixed asset investment after reopening.

	Sample for 2023		Sample for 2012–2022	
	(1)	(2)	(3)	(4)
	Season1_FAI	Semi_FAI	Season1_FAI	Semi_FAI
SUC	0.004***	0.010***	0.000	0.000
	(5.21)	(4.50)	(1.28)	(0.67)
SIZE	0.001***	0.002***	0.001***	0.003***
	(5.65)	(6.63)	(5.48)	(7.53)
LEV	0.000	-0.001	-0.002**	-0.008***
	(0.10)	(-0.20)	(-2.50)	(-3.73)
TOP10	0.011***	0.027***	0.011***	0.023***
	(6.86)	(8.57)	(6.84)	(7.00)
BM	-0.009***	-0.018***	-0.009***	-0.019***
	(-5.39)	(-7.81)	(-10.47)	(-9.69)
TBQ	-0.001*	-0.001*	-0.001***	-0.001***
	(-1.83)	(-1.76)	(-5.44)	(-4.49)
Inhold	-0.003**	-0.009***	-0.003**	-0.007***
	(-2.12)	(-4.60)	(-2.73)	(-3.36)
DISC	-0.000	-0.000	-0.000	-0.001*
	(-0.29)	(-0.30)	(-1.50)	(-1.98)
Constant	-0.010***	-0.025***	-0.011***	-0.037***
	(-3.13)	(-4.89)	(-2.79)	(-5.30)
Location FE	YES	YES	YES	YES
Year FE	NO	NO	YES	YES
Industry FE	YES	YES	YES	YES
N	2654	3271	16344	20052
R2	0.155	0.150	0.107	0.096

Note:

*, **, *** denote significance at the 10%, 5%, and 1% levels, respectively; The robust standard errors shown in parentheses are clustered at the location level. Since the 2012–2022 sample is firm-year data, I include year fixed effects in the third and fourth columns.

To ensure that the positive correlation between SUC and FAI in the first quarterly and semiannual reports in 2023 is not due to the mechanical correlation between SUC and the level of fixed asset investment, I re-regress the sample using the years 2012–2022. As shown in columns (3) and (4), the coefficients on SUC are smaller and insignificant in 2012–2022. The results in Table 4 illustrate that firms with a higher concentration of suppliers are indeed able to ramp up production more quickly when the disaster is over, consistent with positive investor attitudes.

#### 4.3.2. Propensity Score Matching (PSM)

Whether firms centralize their suppliers may be affected by certain unobservable factors, and firms with higher SUC may differ structurally from firms with lower SUC. To control for the bias caused by these potential differences, I select asset size (SIZE), leverage ratio (LEV), accounts payable (AP), fixed asset ratio (FAR), proportion of shares held by the top ten largest shareholders (TOP10), state ownership (SOE) and customer concentration (CUC) as matching variables for PSM. In PSM, I divide the sample into a high SUC group and a low SUC group based on the industry median and match between the two groups based on 1:1 no-return near-neighbor matching. I re-estimate the model (6) using the matched sample. As shown in Table 5, after PSM, the coefficient on SUC remains significantly positive at the 5% level. Table 5 illustrates that main results still robust.

**Table 5:** PSM analysis.

	(1)	(2)	(3)	(4)
	CAR1[–1,1]	CAR1[–1,7]	CAR2[–1,1]	CAR2[–1,7]
SUC	0.009***	0.012**	0.009***	0.012**
	(2.87)	(2.45)	(2.82)	(2.34)
SIZE	0.006***	0.004***	0.003***	0.002**
	(5.84)	(3.76)	(3.41)	(2.47)
LEV	0.002	0.001	0.005*	0.004
	(0.75)	(0.14)	(2.01)	(0.66)
TOP10	0.023***	0.044***	0.021***	0.041***
	(3.94)	(4.06)	(3.59)	(3.96)
BM	-0.033***	-0.054***	-0.032***	-0.045***
	(-6.21)	(-6.43)	(-6.18)	(-5.40)
TBQ	0.001	-0.004***	-0.000	-0.003**
	(0.69)	(-2.80)	(-0.01)	(-2.68)
VOL	-0.206*	-0.396***	-0.174	-0.418***
	(-1.82)	(-3.97)	(-1.51)	(-4.23)
Inhold	-0.005	-0.014*	-0.005	-0.013*
	(-1.52)	(-2.03)	(-1.49)	(-1.91)
DISC	0.004	-0.002	0.004	-0.001
	(1.57)	(-0.76)	(1.55)	(-0.72)
Constant	-0.123***	-0.057***	-0.064***	-0.032
	(-5.63)	(-2.84)	(-2.88)	(-1.64)
Location FE	YES	YES	YES	YES
Industry FE	YES	YES	YES	YES
N	2950	2950	2950	2950
R2	0.140	0.145	0.129	0.130

Note:

*, **, *** denote significance at the 10%, 5%, and 1% levels, respectively; The robust standard errors shown in parentheses are clustered at the location level.

#### 4.3.3. Instrumental Variable (IV) Method

To ensure robustness by removing the effect of endogeneity issues, I perform instrumental variable regressions. Specifically, I re-estimated model (6) by using the industry mean and regional mean of SUC as instrumental variables (IV) based on the heteroskedastic instrumental variables approach proposed by Lewbel [55]. Since Lewbel proposed this method, heteroskedastic instrumental variables method has been widely used [56,57]. The heteroskedasticity-based identification method only needs to satisfy the condition that the error is heteroskedastic to construct a new variable as an IV by utilizing exogenous variables, which can effectively avoid the problems that may be caused by weak instrumental variables in the traditional instrumental variable method. The results are shown in Table 6, where the coefficients of SUC are still significantly positive after controlling for potential endogeneity effects, and the conclusions of this paper are still robust.

**Table 6:** Instrumental variables regression.

	(1)	(2)	(3)	(4)
	CAR1[–1,1]	CAR1[–1,7]	CAR2[–1,1]	CAR2[–1,7]
SUC	0.008**	0.009**	0.008*	0.009*
	(2.05)	(2.12)	(1.92)	(1.92)
SIZE	0.006***	0.004***	0.003***	0.003***
	(6.91)	(6.17)	(4.00)	(4.39)
LEV	0.006***	0.003	0.009***	0.006
	(3.22)	(0.60)	(4.77)	(1.37)
TOP10	0.023***	0.045***	0.021***	0.042***
	(3.94)	(4.26)	(3.53)	(4.10)
BM	-0.028***	-0.047***	-0.027***	-0.037***
	(-7.18)	(-6.31)	(-6.77)	(-5.12)
TBQ	0.003***	-0.002	0.002*	-0.002
	(2.58)	(-1.47)	(1.75)	(-1.34)
VOL	-0.250**	-0.406***	-0.215**	-0.431***
	(-2.41)	(-3.60)	(-2.01)	(-3.90)
Inhold	-0.008*	-0.021***	-0.008*	-0.020***
	(-1.82)	(-2.98)	(-1.80)	(-2.85)
DISC	0.002	-0.004	0.002	-0.004
	(1.19)	(-1.63)	(1.12)	(-1.52)
Constant	-0.135***	-0.125***	-0.078***	-0.098***
	(-7.70)	(-6.21)	(-4.26)	(-4.90)
Location FE	YES	YES	YES	YES
Industry FE	YES	YES	YES	YES
N	4043	4043	4043	4043
R2	0.130	0.133	0.119	0.118

Note:

*, **, *** denote significance at the 10%, 5%, and 1% levels, respectively; The robust standard errors shown in parentheses are clustered at the location level.

#### 4.3.4. Other robustness tests

In addition to the robustness tests described above, I have done other work to ensure the robustness of the results. First, tighter fixed effects are imposed on the regressions. Specifically, I replace the location fixed effects in the main regressions from two-digit coded provinces to four-digit coded cities, and replace all industries with two-digit sub-industries as well. In addition to this I use double clustering robust standard errors for industries and cities. As shown in Table 7, the coefficient on SUC remains significantly positive.

**Table 7:** Tighter fixed effect.

	(1)	(2)	(3)	(4)
	CAR1[–1,1]	CAR1[–1,7]	CAR2[–1,1]	CAR2[–1,7]
SUC	0.009**	0.012*	0.008**	0.011*
	(2.64)	(1.86)	(2.52)	(1.72)
SIZE	0.006***	0.005***	0.004***	0.004**
	(5.58)	(2.73)	(3.41)	(2.00)
LEV	0.001	-0.002	0.004	0.001
	(0.34)	(-0.27)	(1.15)	(0.19)
TOP10	0.022***	0.046***	0.020***	0.043***
	(3.65)	(6.34)	(3.25)	(6.15)
BM	-0.025***	-0.050***	-0.025***	-0.042***
	(-4.15)	(-4.56)	(-3.96)	(-3.85)
TBQ	0.003***	-0.002	0.003**	-0.002
	(3.20)	(-1.27)	(2.39)	(-1.19)
VOL	-0.114	-0.216	-0.081	-0.242
	(-0.41)	(-0.54)	(-0.29)	(-0.62)
Inhold	-0.005	-0.019***	-0.005	-0.018***
	(-1.44)	(-3.56)	(-1.50)	(-3.36)
DISC	0.002	-0.003	0.002	-0.003
	(1.30)	(-1.30)	(1.25)	(-1.28)
Constant	-0.139***	-0.093***	-0.083***	-0.067**
	(-6.47)	(-2.89)	(-3.79)	(-2.07)
City FE	YES	YES	YES	YES
Industry FE	YES	YES	YES	YES
N	3966	3966	3966	3966
R2	0.240	0.216	0.228	0.202

Note:

*, **, *** denote significance at the 10%, 5%, and 1% levels, respectively. robust standard errors in parentheses are double-clustered at the city level and industry level.

Second, change the event window and CAR calculation model. To ensure the robustness of the results, I regress the model (6) using a shorter event window [0,1] and a longer event window [–1,10]. As shown in columns (1)-(4) of Table 8, the coefficients on SUC remain significantly positive, indicating that the previous results are not altered by the event window. In addition, I use the FAMA five-factor model to calculate CAR3[–1,1] and CAR3[–1,7] based on the Fama and French’s study [58]. The coefficient on SUC remains significantly positive as shown in columns (5) and (6) of Table 8.

**Table 8:** Change the event window and CAR calculation model.

	(1)	(2)	(3)	(4)	(5)	(6)
	CAR1[0,1]	CAR2[0,1]	CAR1[–1,10]	CAR2[–1,10]	CAR3[–1,1]	CAR3[–1,7]
SUC	0.007**	0.007**	0.017***	0.015**	0.010***	0.010**
	(2.68)	(2.60)	(3.07)	(2.70)	(3.31)	(2.55)
SIZE	0.006***	0.002***	0.008***	0.003***	0.003***	0.003***
	(9.05)	(3.16)	(8.56)	(2.96)	(3.74)	(4.16)
LEV	-0.003*	0.000	-0.007	0.002	0.009***	0.006
	(-1.70)	(0.15)	(-1.29)	(0.32)	(6.37)	(1.70)
TOP10	0.015***	0.014***	0.054***	0.046***	0.014**	0.021*
	(3.14)	(2.79)	(5.14)	(4.53)	(2.35)	(1.99)
BM	-0.008**	-0.019***	-0.058***	-0.039***	-0.026***	-0.037***
	(-2.36)	(-5.38)	(-5.96)	(-4.00)	(-6.55)	(-4.66)
TBQ	0.004***	0.001	-0.004*	-0.004**	0.002	-0.002
	(4.44)	(1.68)	(-2.04)	(-2.32)	(1.68)	(-1.51)
VOL	-0.458***	-0.324***	-0.482***	-0.505***	-0.312***	-0.718***
	(-5.81)	(-3.80)	(-3.06)	(-3.25)	(-3.13)	(-6.77)
Inhold	-0.003	-0.005*	-0.026***	-0.023***	-0.005	-0.011
	(-1.30)	(-2.02)	(-3.65)	(-3.41)	(-1.17)	(-1.57)
DISC	0.002	0.001	-0.004*	-0.004*	0.003	-0.002
	(1.32)	(0.73)	(-1.93)	(-1.79)	(1.38)	(-1.15)
Constant	-0.151***	-0.044***	-0.152***	-0.037*	-0.060***	-0.033**
	(-12.38)	(-2.98)	(-7.30)	(-1.85)	(-3.08)	(-2.16)
Location FE	YES	YES	YES	YES	YES	YES
Industry FE	YES	YES	YES	YES	YES	YES
N	4043	4043	4043	4043	4043	4043
R2	0.148	0.076	0.120	0.101	0.108	0.106

Note:

*, **, *** denote significance at the 10%, 5%, and 1% levels, respectively; The robust standard errors shown in parentheses are clustered at the location level.

Finally, I change the event date from the reopening announcement date to the implementation date. On December 27, 2022 the Chinese government announced that it would begin lifting the lockdown and isolation policy on January 8, 2023. While in general stock price adjustments tend to occur within a short period of time after the announcement, the market reaction on the implementation day of the policy is also noteworthy. CAR1_IMP and CAR2_IMP for January 8, 2023 were calculated using the market model and the FAMA three-factor model to re-estimate model (6). Panel C of Table 9 shows that the coefficient on SUC remains significantly positive on the policy implementation date.

**Table 9:** The effect of reopening policy implementation.

	(1)	(2)	(3)	(4)
	CAR1_IMP[–1,1]	CAR1_IMP[–1,7]	CAR2_IMP [–1,1]	CAR2_IMP [–1,7]
SUC	0.016***	0.015**	0.016***	0.015**
	(6.15)	(2.52)	(4.98)	(2.16)
SIZE	0.005***	0.008***	0.001	-0.001
	(7.60)	(10.45)	(0.90)	(-1.46)
LEV	-0.005*	-0.024***	0.004	-0.010*
	(-1.70)	(-4.58)	(1.13)	(-1.74)
TOP10	0.022***	0.032***	0.015***	0.022**
	(6.23)	(4.80)	(3.77)	(2.59)
BM	-0.027***	-0.016*	0.000	0.015
	(-5.40)	(-1.72)	(0.00)	(1.53)
TBQ	-0.003***	-0.002	-0.003***	-0.003
	(-2.99)	(-0.98)	(-2.77)	(-1.28)
VOL	0.109	-1.014***	0.022	-1.059***
	(1.23)	(-6.75)	(0.27)	(-6.21)
Inhold	-0.012***	-0.012*	-0.009***	-0.011*
	(-3.69)	(-2.01)	(-2.96)	(-1.81)
DISC	0.000	-0.004	0.001	-0.004
	(0.28)	(-1.53)	(1.00)	(-1.36)
Constant	-0.107***	-0.170***	-0.017	0.041**
	(-6.92)	(-10.02)	(-1.29)	(2.30)
Location FE	YES	YES	YES	YES
Industry FE	YES	YES	YES	YES
N	4043	4041	4043	4041
R2	0.126	0.161	0.064	0.138

Note: *, **, *** denote significance at the 10%, 5%, and 1% levels, respectively; The robust standard errors shown in parentheses are clustered at the location level.

## 5. Cross-sectional analysis

The above analysis finds that the announcement of the reopening policy increases the value of centralized suppliers, with investors showing confidence in firms with high SUC. However, even though all firms face the same reopening shock, there are differences in the magnitude of the impact on different types of firms. Relatively speaking, regional mobility restrictions during the epidemic had a greater impact on some groups of firms that were more affected by supplier, and SUC generates higher production costs and business risks. Then, when the reopening policy is announced, production costs fall more for this group, thus SUC is more valuable. I use three proxies to distinguish between groups of firms that are highly affected by suppliers, namely the industry in which the firm is located, the nature of its ownership, and its commercial credit supply.

### 5.1. Effect of industry

In the early days of the COVID-19 outbreak, the risks resulting from supply chain disruptions showed great variation across industries. After the closure of Wuhan, the stock returns of manufacturing firms suffered a worse decline than other industries due to supply chain risks [10]. The main reason for this phenomenon is that the manufacturing sector is more dependent on the cooperation of its suppliers than other industries. Firms cannot carry out regular production without the timely supply of raw materials. Similarly, I hypothesize that the recovery of non-manufacturing industries at reopening is less sensitive to SUC. In other words, the positive investor response to SUC at the reopening was more noticeable in the manufacturing sector. To test the hypothesis, I classify firms in the second industry into the manufacturing group, and the samples in the first and third industries into the non-manufacturing group. I regress these two subsamples and present the results in Table 10.

**Table 10:** Cross-sectional analysis: Effect of industry.

	(1)	(2)	(3)	(4)	(5)	(6)	(7)	(8)
	CAR1[–1,1]		CAR1[–1,7]		CAR2[–1,1]		CAR2[–1,7]	
	Manufacturing	Non-Manufacturing	Manufacturing	Non-Manufacturing	Manufacturing	Non-Manufacturing	Manufacturing	Non-Manufacturing
SUC	0.015***	0.001	0.012**	0.007	0.014***	0.001	0.011**	0.007
	(3.60)	(0.35)	(2.61)	(0.90)	(3.43)	(0.32)	(2.38)	(0.86)
SIZE	0.006***	0.004***	0.003**	0.007***	0.004***	0.002***	0.002	0.006***
	(4.69)	(7.13)	(2.22)	(3.76)	(2.76)	(3.65)	(1.27)	(3.21)
LEV	0.015***	-0.010**	0.014**	-0.018**	0.018***	-0.006	0.017***	-0.015*
	(3.96)	(-2.53)	(2.53)	(-2.13)	(4.73)	(-1.53)	(3.17)	(-1.74)
TOP10	0.026***	0.014	0.048***	0.035**	0.023***	0.013	0.045***	0.032*
	(4.01)	(1.09)	(4.02)	(2.18)	(3.53)	(1.04)	(3.92)	(1.99)
BM	-0.030***	-0.021**	-0.056***	-0.031**	-0.029***	-0.020**	-0.047***	-0.023*
	(-5.81)	(-2.70)	(-6.78)	(-2.51)	(-5.51)	(-2.61)	(-5.79)	(-1.87)
TBQ	0.003**	0.002	-0.002	-0.002	0.002	0.002	-0.002	-0.002
	(2.21)	(0.95)	(-1.47)	(-0.77)	(1.47)	(0.61)	(-1.35)	(-0.75)
VOL	-0.225	-0.316***	-0.506***	-0.244	-0.181	-0.299**	-0.545***	-0.239
	(-1.58)	(-2.76)	(-3.33)	(-1.44)	(-1.23)	(-2.70)	(-3.65)	(-1.42)
Inhold	-0.010*	0.002	-0.017**	-0.030**	-0.010*	0.001	-0.016**	-0.028**
	(-2.03)	(0.25)	(-2.42)	(-2.30)	(-1.96)	(0.09)	(-2.33)	(-2.19)
DISC	0.002	0.003	-0.007**	-0.001	0.001	0.003	-0.006**	-0.001
	(0.72)	(1.05)	(-2.36)	(-0.35)	(0.58)	(1.12)	(-2.26)	(-0.33)
Constant	-0.140***	-0.097***	-0.050	-0.134***	-0.080**	-0.043**	-0.023	-0.113**
	(-4.39)	(-5.23)	(-1.51)	(-2.82)	(-2.43)	(-2.34)	(-0.72)	(-2.42)
Location FE	YES	YES	YES	YES	YES	YES	YES	YES
Industry FE	YES	YES	YES	YES	YES	YES	YES	YES
N	2765	1278	2765	1278	2765	1278	2765	1278
R2	0.117	0.196	0.118	0.194	0.110	0.172	0.106	0.181

Note:

*, **, *** denote significance at the 10%, 5%, and 1% levels, respectively; The robust standard errors shown in parentheses are clustered at the location level.

As shown in Table 10, the coefficients of SUC for the manufacturing group are more significant than those for the non-manufacturing group both in terms of economic significance and statistical significance. The results in Table 10 as a whole suggest that manufacturing industries need to pay more attention to supply chain management because their profit and loss are closely related to SUC.

### 5.2. Effect of state ownership

The close relationship between state owned enterprises (SOE) and government makes it easier for them to access scarce resources, such as easier credit conditions [59]. This natural advantage weakens the relationship between SUC and business risk in SOE while at the same time weakening the positive relationship between SUC and surplus sustainability [60]. The strong monopoly power of SOE in market competition weakens their reliance on business relationships [61]. Hence I infer that the value-enhancing effect of SUC is more pronounced for non-state-owned enterprises (N-SOE) in the post-disaster period. I categorize the sample into SOE and N-SOE groups according to the nature of ownership of the firms and re-estimate the model (6) in the 2 sub-samples. The results are presented in Table 11.

**Table 11:** Cross-sectional analysis: Effect of state ownership.

	(1)	(2)	(3)	(4)	(5)	(6)	(7)	(8)
	CAR1[–1,1]		CAR1[–1,7]		CAR2[–1,1]		CAR2[–1,7]	
	SOE	N-SOE	SOE	N-SOE	SOE	N-SOE	SOE	N-SOE
SUC	-0.002	0.014***	0.005	0.010**	-0.003	0.013***	0.006	0.009**
	(-0.40)	(3.34)	(0.61)	(2.47)	(-0.55)	(3.28)	(0.69)	(2.23)
SIZE	0.004***	0.006***	0.005**	0.005***	0.002**	0.004***	0.003**	0.003**
	(4.54)	(4.66)	(2.73)	(3.60)	(2.36)	(2.84)	(2.10)	(2.50)
LEV	-0.006	0.012***	-0.000	0.006	-0.002	0.015***	0.002	0.009*
	(-0.98)	(3.71)	(-0.02)	(1.27)	(-0.38)	(4.78)	(0.26)	(1.83)
TOP10	-0.007	0.029***	-0.009	0.047***	-0.005	0.026***	-0.006	0.044***
	(-0.38)	(4.74)	(-0.31)	(4.32)	(-0.25)	(4.25)	(-0.20)	(4.13)
BM	-0.023***	-0.032***	-0.038***	-0.049***	-0.024***	-0.030***	-0.029***	-0.040***
	(-3.09)	(-5.52)	(-3.78)	(-5.16)	(-3.10)	(-5.22)	(-2.93)	(-4.33)
TBQ	-0.002	0.003**	-0.003	-0.002*	-0.002	0.002*	-0.003	-0.002*
	(-0.88)	(2.59)	(-1.18)	(-1.81)	(-1.33)	(1.88)	(-1.10)	(-1.72)
VOL	-0.315*	-0.289**	-0.566*	-0.400**	-0.303	-0.244*	-0.551*	-0.431**
	(-1.72)	(-2.15)	(-1.81)	(-2.51)	(-1.64)	(-1.79)	(-1.77)	(-2.74)
Inhold	0.006	-0.006	0.012	-0.013*	0.003	-0.006	0.009	-0.013*
	(0.36)	(-1.53)	(0.45)	(-2.02)	(0.17)	(-1.49)	(0.36)	(-2.00)
DISC	0.006**	0.001	0.003	-0.006*	0.006**	0.000	0.003	-0.006*
	(2.72)	(0.24)	(0.82)	(-1.94)	(2.62)	(0.17)	(0.95)	(-1.96)
Constant	-0.074***	-0.150***	-0.078*	-0.085***	-0.022	-0.090***	-0.059	-0.055*
	(-4.15)	(-4.76)	(-1.88)	(-2.89)	(-1.18)	(-2.78)	(-1.43)	(-1.93)
Location FE	YES	YES	YES	YES	YES	YES	YES	YES
Industry FE	YES	YES	YES	YES	YES	YES	YES	YES
N	1122	2921	1122	2921	1122	2921	1122	2921
R2	0.203	0.144	0.185	0.132	0.192	0.133	0.173	0.119

Note:

*, **, *** denote significance at the 10%, 5%, and 1% levels, respectively; The robust standard errors shown in parentheses are clustered at the location level.

As shown in Table 11, the coefficients of SUC are more economically and statistically significant in the N-SOE group than in the SOE group. The results in Table 11 as a whole suggest that SUC has a more significant value-enhancing effect on N-SOE in the post-disaster period, and therefore investors react more positively to SUC.

### 5.3. Effect of commercial credit to suppliers

Commercial credit to suppliers (CCS) is a prepayment made by a company to its suppliers for goods and services [62,63]. It reflects the extent to which a company provides liquidity to its suppliers in advance. Higher prepayments indicate weaker bargaining power and higher transaction costs, as firms take on the financial burden of mitigating supply chain risks, especially in times of uncertainty such as pandemics. Firms are in effect providing more liquidity to their suppliers in exchange for assurances of continued delivery, thus increasing their overall financial burden during a crisis. After pandemic restrictions are lifted and the economy fully reopens, uncertainty is removed. Companies no longer needed to offer unusually high prepayments to secure goods. This shift led to a significant reduction in transaction costs, especially for companies that had previously relied on prepayments to mitigate supply chain risk. These companies now benefit from standardized payment terms and no longer need such large financial commitments. As a result, I expect the value-enhancing effect of SUC on high supplier commercial credit firms to become even more pronounced in the post-disaster period.

To test the above hypotheses, I measure CCS as the share of firms’ prepayments to total assets. Based on the median CCS, the sample is divided into a high commercial credit supply group (H_CCS) and a low commercial credit supply group (L_CCS). Model (6) is re-estimated in both subsamples and the results are presented in Table 12.

**Table 12:** Cross-sectional analysis: Effect of commercial credit to suppliers.

	(1)	(2)	(3)	(4)	(5)	(6)	(7)	(8)
	CAR1[–1,1]		CAR1[–1,7]		CAR2[–1,1]		CAR2[–1,7]	
	H_CCS	L_CCS	H_CCS	L_CCS	H_CCS	L_CCS	H_CCS	L_CCS
SUC	0.015***	0.005	0.016**	0.006	0.014***	0.005	0.015**	0.005
	(3.31)	(1.25)	(2.26)	(1.32)	(3.22)	(1.19)	(2.21)	(1.11)
SIZE	0.007***	0.004***	0.006***	0.003***	0.005***	0.002***	0.005***	0.002*
	(5.00)	(6.85)	(4.08)	(3.08)	(3.31)	(3.13)	(3.24)	(1.71)
LEV	0.009	0.003	0.010	-0.005	0.012**	0.006	0.013*	-0.002
	(1.68)	(0.54)	(1.39)	(-0.57)	(2.19)	(1.10)	(1.90)	(-0.20)
TOP10	0.033***	0.014*	0.051***	0.038***	0.030***	0.012	0.048***	0.036***
	(4.11)	(1.89)	(3.70)	(3.28)	(3.84)	(1.57)	(3.49)	(3.25)
BM	-0.027***	-0.029***	-0.043***	-0.052***	-0.026***	-0.028***	-0.033***	-0.044***
	(-3.04)	(-3.66)	(-5.27)	(-3.98)	(-2.94)	(-3.45)	(-4.27)	(-3.31)
TBQ	0.004**	0.002	0.001	-0.005**	0.003	0.001	0.001	-0.005**
	(2.20)	(0.96)	(0.27)	(-2.46)	(1.69)	(0.46)	(0.43)	(-2.34)
VOL	-0.243	-0.236**	-0.437*	-0.332**	-0.203	-0.205*	-0.460**	-0.359**
	(-1.28)	(-2.10)	(-2.04)	(-2.07)	(-1.06)	(-1.75)	(-2.21)	(-2.27)
Inhold	-0.011	-0.005	-0.028**	-0.015*	-0.011	-0.005	-0.026*	-0.014*
	(-1.56)	(-0.89)	(-2.16)	(-2.02)	(-1.53)	(-0.86)	(-2.02)	(-1.99)
DISC	0.001	0.003	-0.002	-0.007**	0.001	0.003	-0.001	-0.006**
	(0.65)	(1.42)	(-0.49)	(-2.12)	(0.66)	(1.31)	(-0.40)	(-2.05)
Constant	-0.166***	-0.093***	-0.127***	-0.035*	-0.110***	-0.034**	-0.102***	-0.009
	(-5.08)	(-6.12)	(-3.62)	(-1.76)	(-3.25)	(-2.23)	(-2.99)	(-0.46)
Location FE	YES	YES	YES	YES	YES	YES	YES	YES
Industry FE	YES	YES	YES	YES	YES	YES	YES	YES
N	2017	2018	2017	2018	2017	2018	2017	2018
R2	0.135	0.148	0.124	0.171	0.122	0.139	0.110	0.156

Note:

*, **, *** denote significance at the 10%, 5%, and 1% levels, respectively; The robust standard errors shown in parentheses are clustered at the location level.

As shown in Table 12, the coefficient of SUC in H_CCS is more significant than that of L_CCS group in terms of both economic and statistical significance. The overall results in Table 12 suggest that the higher the commercial credit of a firm, the more significant the effect of SUC on firm value.

## 6. Moderating effects analysis

In the previous section, I verified that the stock investor reacted positively to the SUC when reopening. However, from the time shock occurs to the time investors react, there is a need for information to be disseminated through the media, especially firm-specific information such as supplier information. I therefore hypothesize that the impact of SUC on CAR when reopening is announced may be influenced by the information medium.

### 6.1. Media attention

Listed companies that are heavily covered by the media are more likely to attract the attention of investors [64,65]. Information in the market is vast and complex, and investor attention becomes a scarce resource that directly affects capital market pricing [66]. If a company receives sufficient attention from investors, its information can be quickly reflected in the stock price when it is hit. Therefore, I believe that media attention may affect stock returns of SUC at reopening. To test this hypothesis, I measure media attention (Media) by the number of times firms appear in financial media reports and re-estimate model (6) by adding the interaction term between Media and SUC. The regression results are presented in Table 13.

**Table 13:** Moderating effects of media attention.

	(1)	(2)	(3)	(4)
	CAR1[–1,1]	CAR1[–1,7]	CAR2[–1,1]	CAR2[–1,7]
SUC	0.009***	0.011***	0.009***	0.010***
	(2.90)	(3.19)	(2.83)	(3.01)
Media	-0.000	0.000	-0.001	-0.000
	(-0.45)	(0.29)	(-0.78)	(-0.08)
SUC# Media	0.012***	0.020***	0.013***	0.018**
	(4.42)	(2.96)	(4.59)	(2.66)
SIZE	0.006***	0.004***	0.004***	0.003***
	(6.13)	(4.62)	(3.90)	(3.47)
LEV	0.007***	0.003	0.010***	0.006
	(3.38)	(0.79)	(4.79)	(1.48)
TOP10	0.021***	0.043***	0.019***	0.041***
	(3.62)	(4.15)	(3.25)	(4.00)
BM	-0.028***	-0.047***	-0.027***	-0.038***
	(-6.93)	(-5.68)	(-6.55)	(-4.66)
TBQ	0.003**	-0.002	0.002	-0.002
	(2.47)	(-1.44)	(1.64)	(-1.30)
VOL	-0.250**	-0.440***	-0.213**	-0.458***
	(-2.57)	(-4.14)	(-2.12)	(-4.39)
Inhold	-0.008*	-0.020***	-0.008*	-0.019**
	(-1.76)	(-2.83)	(-1.77)	(-2.72)
DISC	0.002	-0.005*	0.002	-0.004
	(0.96)	(-1.71)	(0.91)	(-1.60)
Constant	-0.133***	-0.075***	-0.078***	-0.054***
	(-5.98)	(-4.07)	(-3.47)	(-2.98)
Location FE	YES	YES	YES	YES
Industry FE	YES	YES	YES	YES
N	3970	3970	3970	3970
R2	0.133	0.136	0.122	0.121

Note:

*, **, *** denote significance at the 10%, 5%, and 1% levels, respectively; The robust standard errors shown in parentheses are clustered at the location level.

As shown in Table 13, the coefficients of Media # SUC in columns (1)-(4) are all significantly positive at the 5% level, suggesting that media attention amplifies the stock market’s positive response to SUC. The likely reason is that the large amount of media coverage intensifies investor attention and creates a more transparent information environment, so more investors discovery SUC is a valuable signal when the government announces reopening.

### 6.2. Analyst attention

Besides the financial media, another important source of information for investors, especially professional investors, is financial analysts [6,67]. Analysts play a crucial role in asset pricing as information miners and providers. Unlike real-time stock price information, the list of top five suppliers is only published in companies’ annual reports. It is difficult for investors to form a full picture of a company’s operating conditions and make an accurate valuation through fragmented information, especially to react to time-lagged supplier information when shocks occur. However, analysts are the professionals who do the information consolidation, and their specialty is to provide exactly the kind of information that may have been overlooked. Thus, if SUC is indeed a value signal in the post-disaster period, then professional analysts may amplify the value of this signal. To test this hypothesis, I measure analyst attention (Analyst) by the number of analysts tracking firms in 2022 and re-estimate model (6) by adding the interaction term between Analyst and SUC. The regression results are presented in Table 14.

**Table 14:** Moderating effects of analyst attention.

	(1)	(2)	(3)	(4)
	CAR1[–1,1]	CAR1[–1,7]	CAR2[–1,1]	CAR2[–1,7]
SUC	0.010***	0.010***	0.009***	0.009***
	(3.64)	(2.98)	(3.56)	(2.75)
Analyst	0.006***	0.011***	0.005***	0.010***
	(7.51)	(13.62)	(6.54)	(12.60)
SUC# Analyst	0.005**	0.010**	0.006***	0.009**
	(2.56)	(2.67)	(2.86)	(2.51)
SIZE	0.000	-0.006***	-0.001	-0.006***
	(0.43)	(-6.83)	(-1.35)	(-7.60)
LEV	0.014***	0.017***	0.016***	0.019***
	(6.95)	(4.85)	(8.07)	(5.28)
TOP10	0.015**	0.029***	0.013**	0.028***
	(2.68)	(2.85)	(2.41)	(2.78)
BM	-0.011**	-0.014*	-0.012**	-0.008
	(-2.55)	(-1.92)	(-2.59)	(-1.09)
TBQ	0.003**	-0.002	0.002*	-0.002
	(2.71)	(-1.34)	(1.82)	(-1.21)
VOL	-0.321***	-0.543***	-0.281**	-0.555***
	(-3.18)	(-5.14)	(-2.69)	(-5.31)
Inhold	-0.004	-0.015**	-0.005	-0.014**
	(-1.09)	(-2.14)	(-1.16)	(-2.06)
DISC	0.003*	-0.002	0.003	-0.002
	(1.81)	(-0.63)	(1.68)	(-0.60)
Constant	-0.018	0.134***	0.030	0.141***
	(-0.85)	(7.24)	(1.39)	(7.86)
Location FE	YES	YES	YES	YES
Industry FE	YES	YES	YES	YES
N	4043	4043	4043	4043
R2	0.151	0.168	0.138	0.148

Note:

*, **, *** denote significance at the 10%, 5%, and 1% levels, respectively; The robust standard errors shown in parentheses are clustered at the location level.

As shown in Table 14, the coefficients of Analyst # SUC in columns (1)-(4) are all significantly positive at least at the 5% level, suggesting that analyst attention amplifies the stock market’s positive response to SUC. The likely reason for this is that the more analysts tracked the greater the likelihood that potential information will be mined, thus reducing the cost to the investor of obtaining the information. Analysts may collect and organize trivial SUC information, perhaps even detailed lists of suppliers, even if these are not published in the annual report. Analysts communicate valuable supply chain information to the stock market through research reports and other ways, which is reflected in stock prices. As a result, more investors believed that companies with high SUC were more valuable when the government announces reopening.

## 7. Conclusions

Literature on supply chain resilience highlights two contrasting aspects of supply chain centralization: flexibility and vulnerability [4,29]. While firms with centralized supply chains are exposed to significant losses during disruptions, they also benefit from more flexible and efficient inventory adjustments. With the spread of COVID-19, an increasing number of studies have focused on the risk of supply chain vulnerability under uncertain shocks, emphasizing the importance of temporary diversification [40]. However, few studies have explored the sustainable value from the flexibility of centralized supply chains. This study explores how SUC affects stock prices in the post-disaster period with the exogenous shock of China’s reopening announcement, and these results can provide insights into the sustainability of firms in the post-disaster period.

The empirical results suggest that SUC has a positive impact on CAR during the reopening period, which still holds after a series of strict robustness tests. The robustness tests also find that SUC does contribute to production recovery after the epidemic, a contribution that was not present before reopen policy, validating the rationality of investors’ attitudes towards SUC. It is worth noting that this result is the exact opposite of Cheng et al.’s findings—their study states that investors perceived SUC as a risk in the early stages of the epidemic [10]. The key reason for the difference between the two papers is the changing market environment. In a volatile environment (as Cheng et al. (2022) focuses on the period of disruption) the risks of supply chain centralization outweigh the benefits, whereas when the crisis is over (as this study focuses on the period of reopening) firms with centralized supplier relationships can benefit from simplified coordination and reduced transaction costs. Investor sentiment shifted from viewing SUC as a risk in the early stages of the epidemic to viewing it as a value enhancer in the post-disaster period. This highlights the dynamic nature of supply chain strategies in response to changing economic conditions.

In addition, I conducted a cross-sectional analysis to test several attributes that influence market reactions. The cross-sectional analysis shows that positive investor attitudes towards SUC are only present in the manufacturing sector, which is consistent with the fact that the manufacturing sector is more supplier-dependent than the agriculture and service sectors. Second, non-state-owned enterprises and those that provide more commercial credit to their suppliers can also benefit from SUC. This is because state-owned enterprises and firms that provide lower commercial credit to suppliers have more bargaining power and less risk of damage or disruption in a disaster. As a result, post-disaster benefits and supply chains are also smaller and positive policy shifts do not significantly reduce their transaction costs.

Finally, I focus on the moderating effect of the information channel on SUC. The analysis finds that investors’ attitudes towards SUC are influenced by media attention and analysts’ attention, suggesting that information intermediation amplifies the positive effects of SUC. This suggests that the financial media and financial analysts play an essential function, taking on the role of information mining and transmission in the transfer of information from corporate finance to the stock market.

Overall, the study has practical economic implications. First, the study provides a theoretical contribution to firms’ supply chain allocation decisions. literatures such as Cheng et al. and Wang et al. focus on the risk of supply chain disruptions, and they warn that firms should be alert to the risks of centralization [8,10]. However, as transaction cost theory suggests, there are many benefits to centralization, and many firms also choose to establish close deals with a small number of suppliers over the long term [3–5]. This study provides a theoretical explanation for the behavior of these firms. I find that when the epidemic is over, the market is generally bullish on firms with high SUC, validating the positive effects of centralization. Thus, appropriately increasing supply chain concentration may contribute to firms’ sustainability, especially during the post-disaster period when market demand expands. Second, the study provides a decision support for investors’ investment behavior. Supply chain information has become an important tool for investors’ valuation, and assessing the value of a company based on the operating conditions of upstream and downstream firms is conducive to investors’ excess returns. However, investment managers need to be aware that the same factors in different situations may cause different or even completely opposite results on valuation.

## Supporting information

S1 File Code and data. (ZIP)
